# A Novel Mutation in TSC2 Gene: A 34-Year-Old Female with Pulmonary Lymphangioleiomyomatosis with Concomitant Hepatic Lesions

**DOI:** 10.1155/2018/5928231

**Published:** 2018-02-15

**Authors:** Mehdi Nadiri, Mortaza Raeisi, Seyed Ali Mousavi Aghdas

**Affiliations:** ^1^Pulmonology Department, Imam Reza Hospital, Tabriz University of Medical Sciences, Tabriz, Iran; ^2^Hematology & Oncology Research Center, Tabriz University of Medical Sciences, Tabriz, Iran; ^3^Students' Research Committee, Tabriz University of Medical Sciences, Tabriz, Iran

## Abstract

Tuberous sclerosis complex (TSC) is an autosomal dominant disease resulting from mutation(s) in TSC1 or TSC2 genes. TSC is associated with the formation of hamartomas in the brain, heart, eyes, skin, kidneys, and lymphangioleiomyomatosis (LAM) of the lungs. LAM is almost restricted to women in reproductive age. Different mutations in TSC1 and TSC2 genes have been reported in the literature. Here, we present a female patient with TSC-LAM with a novel mutation in TSC2 gene. The patient also had multiple hepatic angiomyolipomas, which is a relatively less-reported manifestation of the disease. The impact of this mutation on the pattern of disease presentation and response to treatment is not clear yet.

## 1. Case Presentation

**Figure 1:**
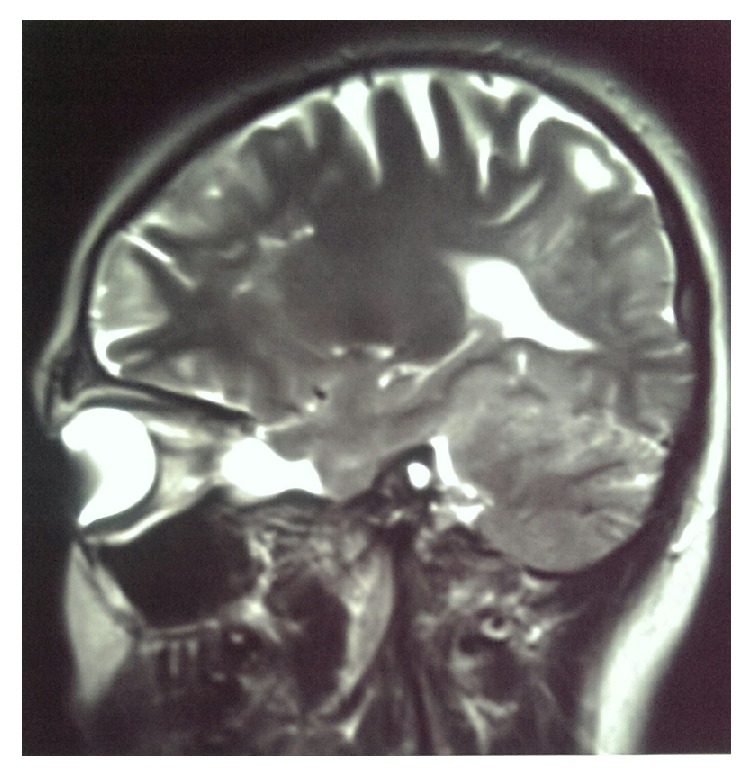
T2 weighted MRI of the brain in sagittal section, showing low-signal subependymal lesion beneath lateral ventricles along with bilateral supratentorial subcortical flair hyperintense signal lesions.

**Figure 2:**
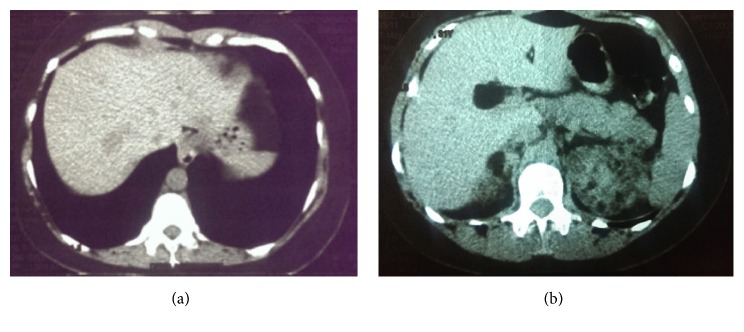
(a) CT cut from the abdomen showing multiple hypodense lesions in the liver with the largest lesion in the right lobe. (b) Lower cut showing multiple angiomyolipomas in the upper pole of the right and left kidney, which have interrupted the normal size, shape, and density of the organ. The Hounsfield values of the lesions are similar to that of the subdermal fat tissue.

**Figure 3:**
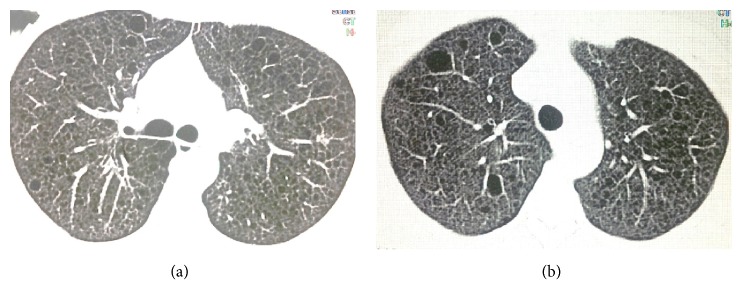
HRCT of the chest, showing diffuse cystic changes distributed equally in all regions of both lungs.

The case is a 34-year-old woman, with the chief complaint of tightness of breath. Dyspnea had begun gradually 3 years before, was progressive in nature, and was not concomitant with cough, sputum, hemoptysis, pleuritic chest pain, wheeze, or weight loss. The patient had no history of smoking and her occupation did not expose her to environmental pollution or toxins. The patient was exposed to tuberculosis (TB) by her mother, who had inactive old TB. The patient had been previously referred to several other clinicians and, with diagnosis of asthma, was under treatment with multiple sprays. In spite of regular use of the sprays, her symptoms had not relieved. We asked for prior medical histories and patient claimed that she had history of epileptic attacks and was under treatment for her condition with valproate. In physical examination, lungs had generalized reduced respiratory sounds and were hyperresonant in percussion. Patient had hypomelanotic facial macules with fibrous facial plaques. Spirometry test conducted at our visit showed moderate obstructive ventilatory impairment (FEV1/FVC = 84%, FEV1 = 62% of the predicted values). The obstruction showed no significant response to bronchodilators (change in FEV1 after bronchodilator = 9% of the FEV1). Patient had no new brain magnetic resonance imaging (MRI) and the etiology of attacks was not clear. We recommended a brain MRI, which revealed multiple low-signal subependymal lesions less than 1 cm in size, beneath both right and left lateral ventricles along with multiple bilateral supratentorial subcortical flair hyperintense signal lesions (Figure 1). Electroencephalography was also obtained and no epileptic discharges were seen at the time. Also considering the history of TB exposure and her resistant dyspnea, a high-resolution computerized tomography (HRCT) of the chest was recommended; as a result, diffuse atelectasis of bronchioles in both lungs was seen along with multiple thin-walled cystic lesions distributed equally in all pulmonary zones (Figure 3). Our differential diagnoses for this finding were lymphangioleiomyomatosis, Birt-Hogg-Dube syndrome, pulmonary Langerhans' histiocytosis, lymphoid interstitial pneumonia, amyloidosis, follicular bronchiolitis, and pulmonary adenocarcinoma. In abdominal cuts of a chest CT some very suspicious lesions were also seen in the liver and kidneys, which had the Hounsfield values of the subdermal fat tissue (Figure 2). Ultrasonography of the kidneys and liver reported significantly increased heterogenous echogenicity of the renal parenchyma (without any distinct lesions) and multiple echogenic lesions in the right and left lobes of the liver with maximum diameter of 40 mm in the right lobe. Although other differential diagnoses were also possible, LAM-TSC was the most probable of them because of the pattern of the findings from chest HRCT, brain MRI, dermatological findings, and lesions in the liver and kidneys.

According to European Respiratory Society guidelines for the diagnosis and management of LAM, the diagnosis can be made clinically with the combination of findings from high-resolution CT and patient's history (angiomyolipoma, lymphangiomyolipoma, chylothorax or abdominal chylous effusion, definite or probable TSC) and/or by pathological diagnosis made by tissue biopsy. There is no obligation to redo a biopsy if the other criteria are met [1]. Because none of the lesions were previously confirmed to be TSC associated, we asked the patient to give consent for an ultrasonography-guided kidney biopsy. However, she rejected to give consent after understanding the method of the procedure. Later, we recommended a genetic test to confirm this diagnosis and rule out Birt-Hogg-Dube syndrome. TSC1 and TSC2 gene mutation analysis was performed and, as a result, a novel variant mutation was detected in TSC2 gene as follows: c.3599G>C; p.Arg1200Pro; Het. The method for this analysis included using a Nimblegen chip capturing TSC1 and TSC2 genes followed by Next Generation Sequencing. Definite diagnosis of LAM-TSC was made. To the best of our knowledge, this mutation has not been reported in the literature, and its impact on pattern of disease presentation and response to treatment is not clear yet. A transthoracic echocardiography was taken to rule out the cardiac burden of the disease. Normal function and anatomy of the heart were seen, and no rhabdomyomas were detected in the heart. At this time, we decided to put the patient on sirolimus (1 milligram twice daily) as the main medication for her systemic and pulmonary symptoms. Tests before starting the treatment showed border line level of alanine transferase (ALT) 32 U/L (upper limit of normal for women <31 U/L). The patient was strongly recommended to avoid pregnancy. More than one year after starting sirolimus, patient's liver function tests are nearly left intact (last test results: ALT 45 U/L, aspartate aminotransferase (AST) 34 U/L (ULN for women 31 U/L), alkaline phosphatase 309 U/L (ULN for women <306 U/L), total bilirubin 1.02 mg/dl, urea 34 mg/dl, creatinine 0.83 mg/dl). Patient has not developed hyperlipidemia or any hematologic disorders. In follow-up visits, a body plethysmography was ordered and results are as follows: FEV1/FVC = 59.4%, FEV1 = 64.4%, TLC = 75.8%, RV = 50.6% of the predicted values. It appears that pulmonary function has remained stable. No events of pneumothorax or epileptic attacks have occurred during this period. In the latest ultrasonography, shrinkage of the tuberous lesions in the liver is seen. Largest echogenic lesion in the right lobe of the liver (as mentioned above) has shrunk to diameter of 34 mm. Unfortunately, the size and echogenicity of the renal parenchyma has not changed significantly. Facial lesions have diminished, and patient no longer visits dermatologist for cosmetic purposes.

## 2. Discussion

Tuberous sclerosis complex (TSC) is an autosomal dominant disease resulting from mutation(s) in TSC1 (coding hamartin) or TSC2 (coding tuberin) genes. These genes are classified as tumor suppressor genes [2]. Variety of mutations in TSC1 and TSC2 genes have been mentioned in the literature [3, 4]. TSC is associated with the formation of hamartomas in different organs including cortical tubers, subependymal nodules and subependymal giant-cell astrocytoma in the brain (causing seizures, mental retardation), multiple retinal nodular hamartomas, hypomelanotic macules, facial angiofibromas, shagreen patch and periungual fibromas of the skin, cardiac rhabdomyomas, renal angiomyolipomas (AMLs), and pulmonary lymphangioleiomyomatosis (LAM) [5]. This process causes progressive dysfunction of the involved organs, and current studies have shown the benefit of inhibition of mammalian target of rapamycin (mTOR). Sirolimus and everolimus both have shown promising outcomes in stabilizing and even improving the forced vital capacity and residual volume, reducing serum levels of VEGF-D, and shrinking the AMLs of the kidney [6, 7].

Serum VEGF-D level is correlated with the severity of lung involvement, measured as LAM CT grade, and is almost always higher in LAM patients, although it is slightly lower in pulmonary cystic lesions-only patients. A prospective analysis of the MILES trial demonstrated reduce in VEGF-D levels following treatment with sirolimus and about 134 ml difference in FEV_1_ for each one-unit increase in VEGF-D baseline levels.

Here, we presented a female TSC-LAM patient with a novel mutation, detected in TSC2 gene. The patient also had multiple hepatic AMLs, which is a less-reported manifestation of the disease and may be due to her particular TSC2 mutation [8]. Since the impact of this mutation on the pattern of disease presentation and response to treatment is not clear, we decided to report this case.
